# Superpixel-based segmentation of muscle fibers in multi-channel microscopy

**DOI:** 10.1186/s12918-016-0372-2

**Published:** 2016-12-05

**Authors:** Binh P. Nguyen, Hans Heemskerk, Peter T. C. So, Lisa Tucker-Kellogg

**Affiliations:** 1 Centre for Computational Biology, and Program in Cancer and Stem Cell Biology, Duke-NUS Medical School, Singapore, 169857 Singapore; 2 BioSystems and Micromechanics (BioSyM) Singapore – MIT Alliance for Research and Technology, Singapore, 138602 Singapore; 3 Department of Mechanical Engineering, Massachusetts Institute of Technology, Cambridge, MA, 02139 USA

**Keywords:** Superpixel, Segmentation, Muscle fibers, Confetti fluorescence, Multi-channel microscopy

## Abstract

**Background:**

Confetti fluorescence and other multi-color genetic labelling strategies are useful for observing stem cell regeneration and for other problems of cell lineage tracing. One difficulty of such strategies is segmenting the cell boundaries, which is a very different problem from segmenting color images from the real world. This paper addresses the difficulties and presents a superpixel-based framework for segmentation of regenerated muscle fibers in mice.

**Results:**

We propose to integrate an edge detector into a superpixel algorithm and customize the method for multi-channel images. The enhanced superpixel method outperforms the original and another advanced superpixel algorithm in terms of both boundary recall and under-segmentation error. Our framework was applied to cross-section and lateral section images of regenerated muscle fibers from confetti-fluorescent mice. Compared with “ground-truth” segmentations, our framework yielded median Dice similarity coefficients of 0.92 and higher.

**Conclusion:**

Our segmentation framework is flexible and provides very good segmentations of multi-color muscle fibers. We anticipate our methods will be useful for segmenting a variety of tissues in confetti fluorecent mice and in mice with similar multi-color labels.

**Electronic supplementary material:**

The online version of this article (doi:10.1186/s12918-016-0372-2) contains supplementary material, which is available to authorized users.

## Background

Cells can be genetically engineered with fluorescence genes that are inherited when the cell divides, meaning the descendents use the genes to continuously synthesize fluorescent proteins. This allows a form of biology experiment called “lineage tracing” to see the long-term impact of specific populations of labelled cells. The labelled cells can be bred into an animal from birth, can be injected, transplanted, etc.

Snippert et al. [1] developed a 4-color “confetti” transgene for labelling stem cells. The confetti transgene exploits genetic recombination to achieve a random choice of color (red, yellow, cyan, green) in each stem cell. When a stem cell is induced to divide (whether by natural turnover or by injury), each daughter cell expresses the same color as its ancestor. This creates a patch of homogeneous color in the regenerated tissue. Regenerated cells of different color must have originated from different stem cells. Confetti fluorescence and other multi-color cell labelling strategies are useful for tracking regeneration in adult mice, for evaluating the potency of stem cells in vivo, or for judging the effectiveness of stem cell therapies.

**Fig. 1:**
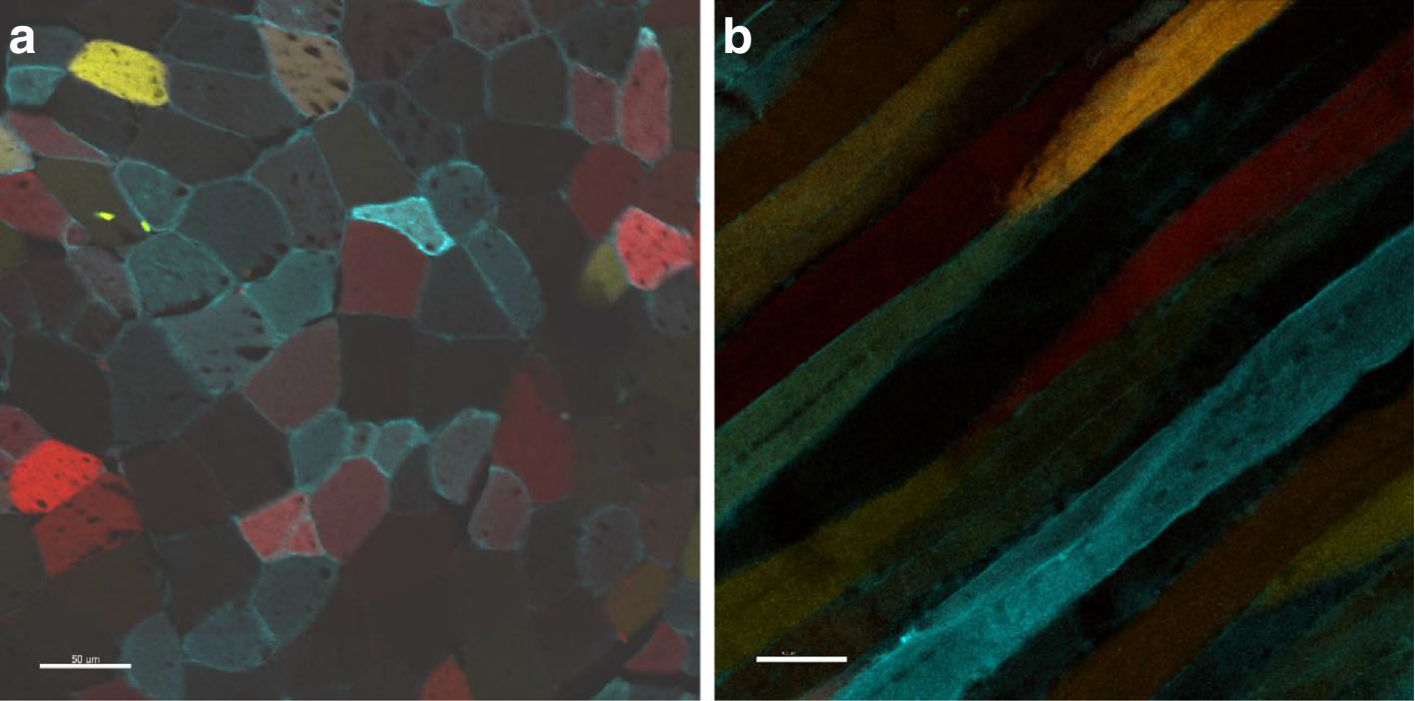
A cross-section (**a**) and a lateral-section (**b**) of regenerated muscle fibers from confetti-fluorescent mice. A five-month-old male (**a**) and female (**b**) mice were injected with 100 *μ* *g*/*g* tamoxifen on 5 consecutive days to achieve transgene recombination. Ten days after the last tamoxifen injection, muscle injury was induced by injecting the tibialis anterior with 50 *μ* *l* 10 *μ* *M* cardiotoxin. Sixteen days after injury mice were sacrificed, the tibialis anterior was fixed and frozen, and 10 *μ* *m* sections were cut. Scalebars are 50 *μ* *m*

In this project we address the analysis of multi-color stem cells after muscle regeneration. Skeletal muscle is a highly regenerative tissue in which each mature muscle cell is a long thin fiber with many nuclei. This muscle fiber is surrounded by a basal lamina, which gives the muscle fiber its firmness during contraction. Muscle-resident stem cells, called satellite cells, are located between the muscle fiber and the basal lamina. If the muscle fiber is severely damaged, it will become necrotic and induce an immune reaction. This activates the satellite cells, which migrate to the injured area and divide into a set of myoblasts. The myoblasts each have one nucleus, but upon differentiation they fuse together in a linear configuration to generate a multi-nucleated myotube or myofiber. The myoblasts can also fuse to pre-existing or partially-damaged fibers [2]. Figure 1 shows a cross-section and a lateral-section of regenerated muscle fibers from confetti-fluorescent mice.

**Fig. 2:**
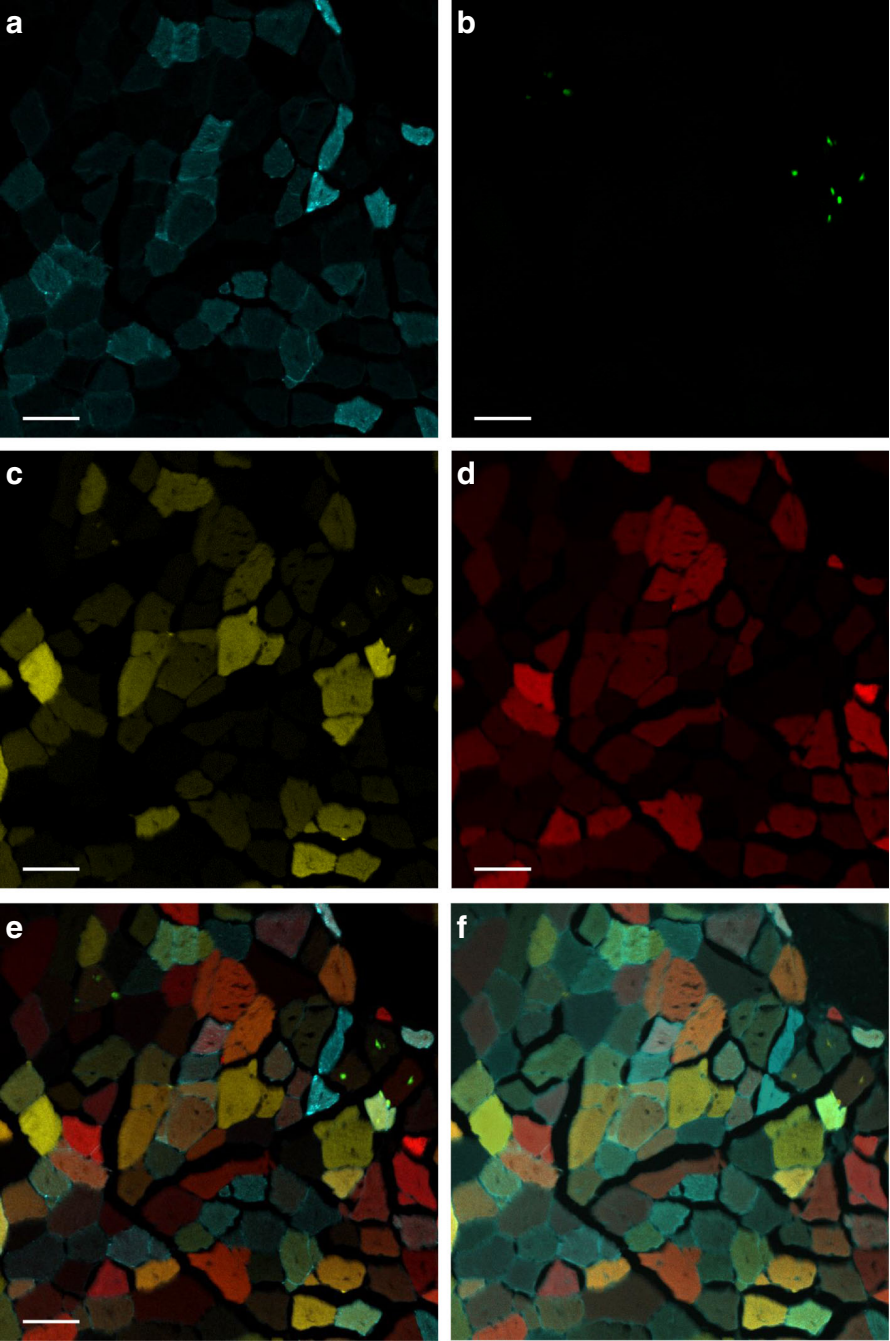
A multi-channel microscopy image of muscle fibers. (**a**) *Cyan* channel; (**b**) *Green* channel; (**c**) *Yellow* channel; (**d**) *Red* channel; (**e**) Composite color image from all the four channels; (**f**) Composite color image from *cyan*, *yellow* and *red* channels after preprocessing. This section is from the same muscle of the cross-section in Fig. 1 a. Scalebars are 50 *μ* *m*. Each channel is a 12-bit image of 1024 × 1024 pixels

Cells with multiple fluorescent proteins are imaged using multi-channel microscopy (such as confocal or two-photon imaging). For example, the image datasets used in our project include four channels: cyan, green, yellow and red (Fig. 2). Each of the fluorescent proteins emitting those colors was excited by a laser at its respective excitation wavelength. The light emitted from the sample contains autofluorescence, so the light is passed through a band-pass filter specific for each fluorescent protein, before detection with a camera. The resulting images show which muscle fibers are positive for which fluorescent proteins. Because muscle fibers are multinucleated cells, an overlay of the four colors can show muscle fibers positive for more than one color.

To analyze the regeneration results, the images must be segmented. A cursory glance at a composite color image in Fig. 2 e may lead to the conclusion that this segmentation is similar to the segmentation of real-world color images. However, our problem is different or more difficult in some ways as follows. In our images, there is extensive contact or overlap between objects (squeezed together), meaning that contour-closing (e.g., in snake- or level sets-based methods) does not work very well for segmentation. We also cannot re-use methods that combine object recognition with segmentation unless we develop domain-specific object models. In addition, many of the boundaries are blurry; some of the objects are in the process of fusing; and there is tremendous variation in the fiber brightness. Using a conventional color difference measure may not be appropriate in our problem since color similarity in multi-channel imaging is different from color similarity in a normal spectrum of visible light. Furthermore, the images have random noise and non-random artifacts including optical aberration from the imaging device; damaged tissue or fracture planes during sample preparation; and ice crystals which cause small empty holes in the image. The ice crystals often have clear boundaries but they should be omitted from the segmentation results. Finally, the four colors of the confetti construct are in different locations within the cell. Green is located in the nucleus, yellow and red are in the cytosol and cyan is on the membrane. In mature muscle fibers, the membrane becomes a sarcolemma with many invaginations. As a result, cyan fluorescence can be seen inside the muscle fibers as well as along the cell-cell edges.

We propose a novel method called SLIC-MMED (simple linear iterative clustering on multi-channel microscopy with edge detection) which uses superpixels for the segmentation of muscle fibers in muli-channel microscopy. A superpixel is a perceptual grouping of neighboring pixels that aligns better with image edges than a rectangular patch [3]. Superpixels are widely used in numerous applications in computer vision including image segmentation. Among existing superpixel generation methods [4], simple linear iterative clustering (SLIC) [5] was chosen for our project because of its effectiveness, scalability and speed. However, SLIC needs to be modified to adapt to our problem. Nuclei are orders of magnitude smaller than mature muscle cells, but when colored green they have a very strong color boundary. This difference in the scale of color change (due to scale difference in underlying objects) could confound the superpixel generation. So we first remove the nuclei. A simple method for segmenting the nuclei turns out to be extremely accurate by using domain-specific information (rounded or ellipsoidal morphology, green color).

To make existing image processing algorithms useful for our domain, we had to perform several modifications: (1) forking different channels to different methods, based on an object model for nuclei, (2) introducing an enhanced superpixel method named *simple linear iterative clustering on multi-channel microscopy with edge detection* (SLIC-MMED), and (3) developing a semi-automatic segmentation framework based on superpixels that can produce very good results for our problem. We believe that all three of these modifications will be useful for other forms of multi-channel cell microscopy, for non-neuronal eukaryotic cell types.

## Methods

### Overview of the framework

**Fig. 3:**
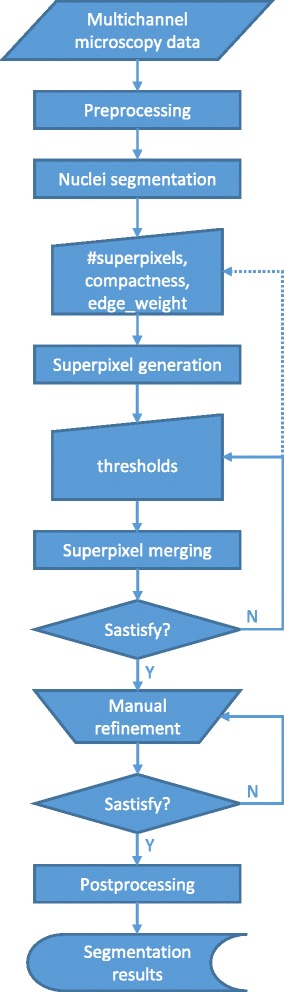
Processing steps in the proposed framework

**Fig. 4:**
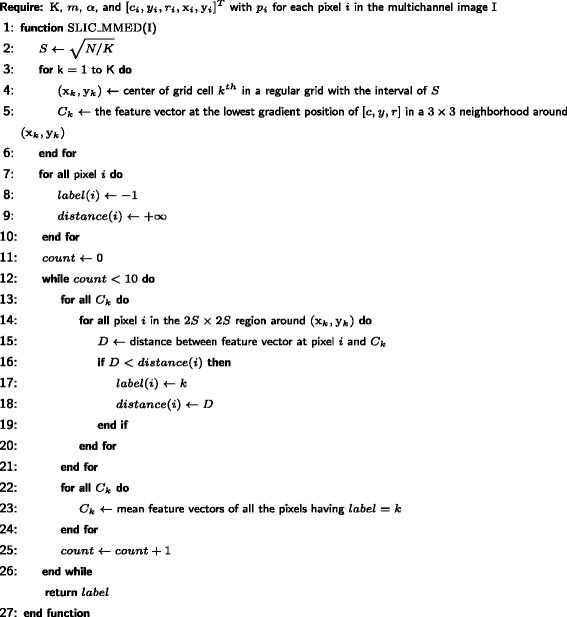
Simple Linear Iterative Clustering on Multichannel Microscopy with Edge Detection (SLIC-MMED) Algorithm

**Fig. 5:**
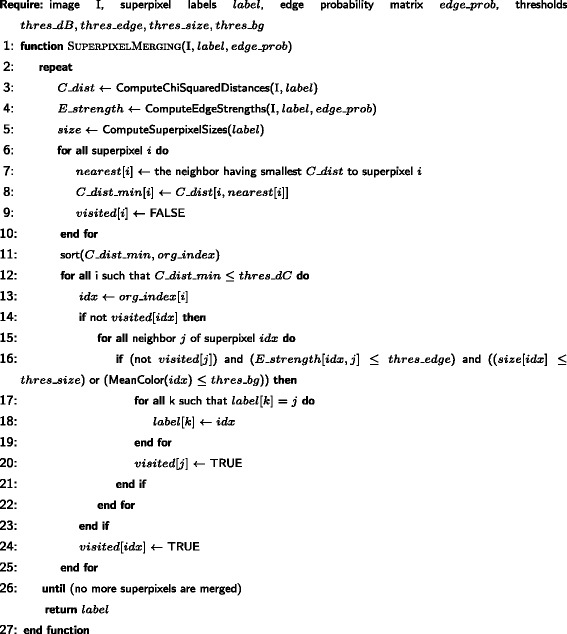
Superpixel Merging Algorithm

An overview of our framework is shown in Fig. 3. First, each channel undergoes intensity normalization and noise reduction filtering. Then the green channel is processed to extract the nuclei. The remaining channels (cyan, yellow and red) are used to generate superpixels according to our SLIC-MMED algorithm (Fig. 4). Next, an automatic superpixel merging algorithm (Fig. 5) is executed to merge a subset of the generated superpixels to form the muscle fibers. The superpixel generation and/or this superpixel merging step can be performed repeatedly with different user-defined parameters until users are satisfied or until termination criteria are reached. After that, the resulting superpixels and merged regions can be further revised through a user-friendly graphical user interface (GUI). Lastly, this segmentation is combined with the nuclear segmentation to form the final result. In this section, we use the dataset shown in Fig. 2 to describe our proposed framework. The primary steps in our framework are detailed below.

### Preprocessing and nuclear segmentation

The first preprocessing is to normalize each channel to the same range, e.g., [0, 255]. Another optional preprocessing step is to apply a noise reduction filter, e.g., median filter, to each channel in cases of noisy images. Figure 2 f shows an example of a composite color image from cyan, yellow and red channels after preprocessing.

**Fig. 6:**
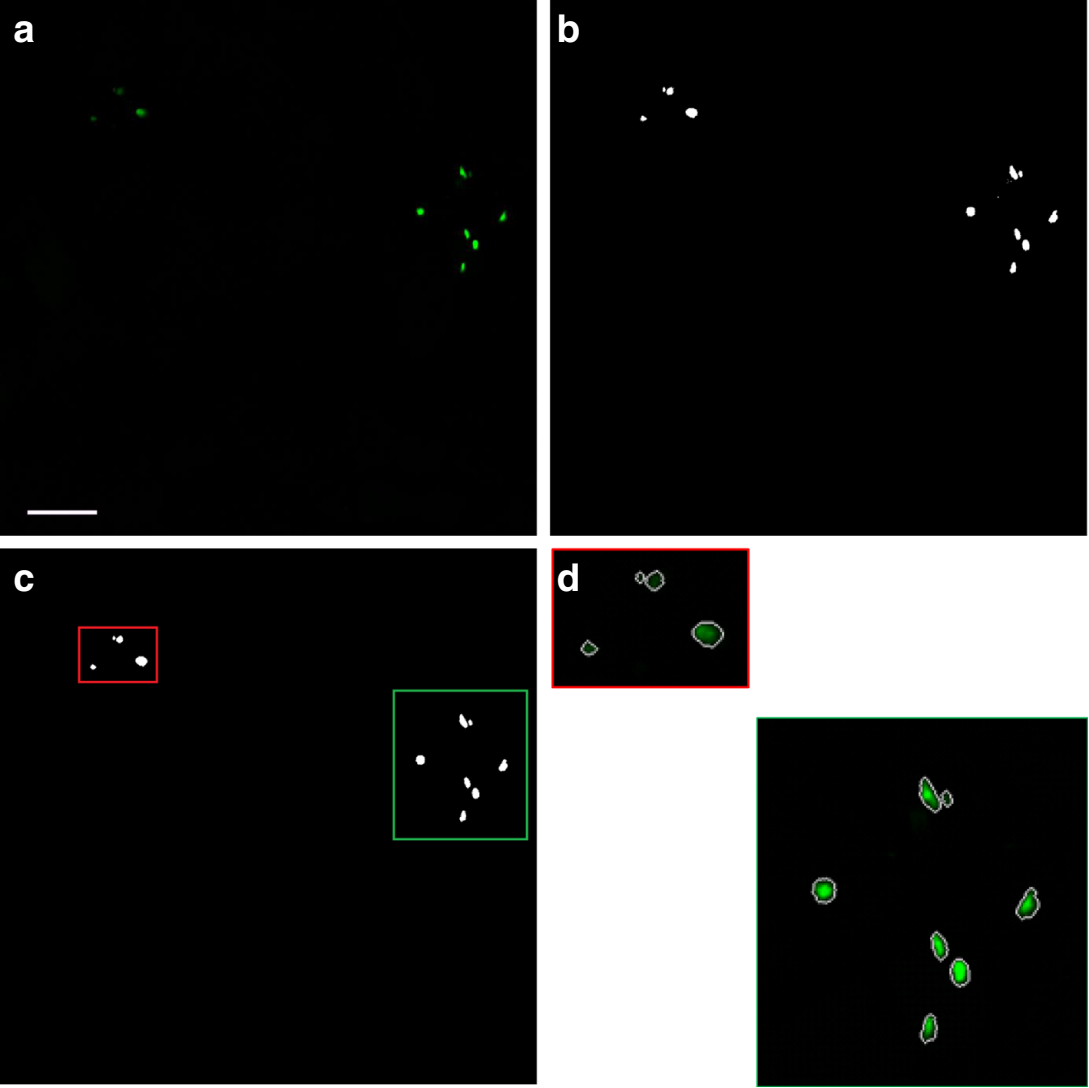
Nuclear segmentation. (**a**) *Green* channel after preprocessing (scalebar is 50 *μ* *m*); (**b**) After image thresholding; (**c**) Using morphological techniques to remove noise; (**d**) Segmented nuclei (enlarged for detail)

In microscopy, cell nuclei are often the easiest morphological features to identify (whether by eye or by algorithm), and many microscopy protocols include nuclear staining, or more recently, fluorescent proteins genetically engineered for nuclear localization. Because each fluorescent tag corresponds to one (or at least one) channel, we can analyze channels individually, based on this knowledge of the underlying signal sources. In other words, we can analyze the green channel for nucleus-like objects. By using image thresholding and morphological techniques, the nuclei in the dataset shown in Fig. 2 can be segmented using only the green channel as illustrated in Fig. 6.

### SLIC-MMED for superpixel generation

The original SLIC algorithm operates on color images in the CIELAB color space with one input parameter is *K*, the desired number of superpixels. Each pixel is represented by a 5-dimensional feature vector, [*l,a,b*,x,y]^*T*^, containing 3 color components and 2 pixel coordinates. At the initialization step, *K* initial cluster centroids *C*_*k*_=[*l*_*k*_,*a*_*k*_,*b*_*k*_,x_*k*_,y_*k*_]^*T*^ are sampled on a regular grid with the interval of $S = \sqrt {N/K}$, where *N* is the number of pixels. To reduce the chance of centering a superpixel on an edge or on a noisy pixel, each centroid is moved to the lowest gradient position in a 3×3 neighborhood. Next, in the iteration step, each pixel is associated with its nearest centroid. In order to speed up the algorithm (compared with *k*-means clusturing), the size of the search space is reduced to a region proportional to the superpixel size. Here, for each cluster centroid *C*_*k*_, only the pixels in the 2*S*×2*S* region around *C*_*k*_ are evaluated, meaning that if the distance from a pixel to *C*_*k*_ is less than the distance from that pixel to its current associated centroid, then the associated centroid will be changed to *C*_*k*_. Once all the pixels have been assigned to their nearest centroids, an update process adjusts each centroid to be the mean feature vector of all the pixels belonging to the corresponding cluster. In practice, repeating this iteration step 10 times is sufficient for most images. Finally, a postprocessing step assigns all disjoint pixels (if any) to nearby superpixels.

In SLIC, the distance *D* between two pixels *i* and *j* is a combination of two distances, *d*_*c*_ and *d*_*s*_, representing color proximity and spatial proximity, respectively, as below:

1 $$  \begin{array}{l} d_{c} = \sqrt {(l_{i} - l_{j})^{2} + (a_{i} - a_{j})^{2} + (b_{i} - b_{j})^{2} }, \\ d_{s} = \sqrt {(\mathrm{x}_{i} - \mathrm{x}_{j})^{2} + (\mathrm{y}_{i} - \mathrm{y}_{j})^{2} }, \\ D = \sqrt {\left(d_{c} / m \right)^{2} + \left(d_{s} / S \right)^{2} }, \\ \end{array}  $$

where the *compactness* *m* is used to to weight the relative importance of color similarity versus spatial proximity. When *m* is large, spatial proximity is more important, and the resulting superpixels are more compact. In contrast, a small value of *m* leads to superpixels that are less regular in size and shape; however, since in this case color proximity is more important, the resulting superpixels follow the image boundaries more closely.

Since the input data in our project are multichannel microscopy images, not real-world color images, the feature vector needs to be modified. Instead of using 3 CIELAB color components (*l,a,b*), we use each image channel as a component of the feature vector. Since the green channel represents only nuclei, it is discarded from the feature vector and processed separately as described in the previous subsection. In short, the feature vector representing each pixel in our data is [*c,y,r*,x,y]^*T*^ which consists of the pixel intensities in the 3 channels cyan, yellow, red, and the pixel coordinates (x,y), respectively.

**Fig. 7:**
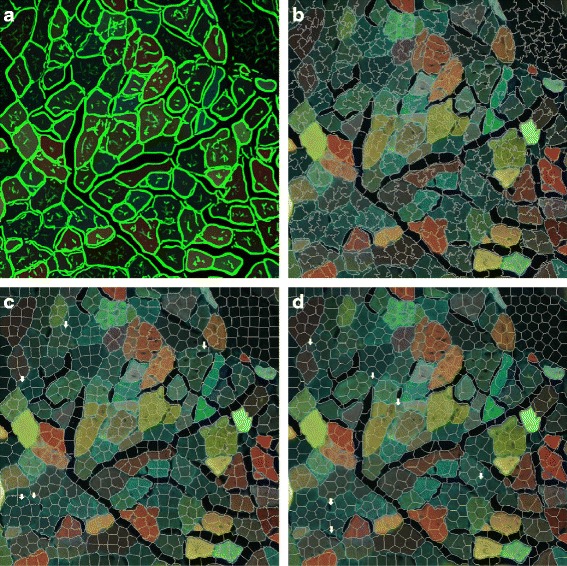
Superpixel generation using different algorithms. (**a**) Computed edge map. The segmentation results from (**b**) our method, SLIC-MMED; (**c**) SLIC; (**d**) VCells. *White arrows* indicate poorly segmented superpixels

In the superpixel method benchmarking, boundary recall is used to measure the fraction of the ground truth edges falling within at least two pixels of a superpixel boundary. A good superpixel segmentation should adhere to object boundaries, meaning that it should produce a high boundary recall. Although SLIC demonstrates very good boundary recall performance for real-world color images [4, 5], this is not the case for our datasets as shown in Fig. 7 c. To overcome these problems, we propose to integrate an additional score *d*_*e*_ into the pixel distance measure. *d*_*e*_ represents the presence of edges between two pixels, suggesting the likelihood that an object boundary falls between the two points. Before starting superpixel generation, an edge detection algorithm is executed to compute a value *p*_*i*_ for each pixel *i*, indicating its probability of being on an edge (boundary). Then the distance *d*_*e*_ between two pixels *i* and *j* is calculated as the maximum edge probability over all the pixels lying on the line connecting pixel *i* and pixel *j*. The new distance is calculated as below.

2 $$  \begin{array}{l} d'_{c} = \sqrt {(c_{i} - c_{j})^{2} + (y_{i} - y_{j})^{2} + (r_{i} - r_{j})^{2} }, \\ d_{s} = \sqrt {(\mathrm{x}_{i} - \mathrm{x}_{j})^{2} + (\mathrm{y}_{i} - \mathrm{y}_{j})^{2} }, \\ d_{e} = \max\limits_{\forall t \in line(i,j)} {p_{t}}, \\ D' = \sqrt {\left(d'_{c} / m \right)^{2} + \left(d_{s} / S \right)^{2} + \alpha \times \left(d_{e} \right)^{2} }, \\ \end{array}  $$

A wide range of edge detection algorithms would be appropriate for computing the edge probabilities *p*_*i*_, and we chose a detector based on the photometric invariance theory and tensor-based features [6]. Figure 7 a shows an edge map generated using that edge detector. Note that if no edge detection were involved (i.e., if *p*_*i*_=0 ∀*i*) then the revised distance measure *D* ^′^ (Eq. 2) would reduce to the original SLIC distance measure *D* (Eq. 1). The new algorithm is presented in Fig. 4.

### Automatic superpixel merging

After superpixel generation, all the superpixels within each muscle fiber need to be merged together to form the muscle fiber boundary. The similarity measure used in our method, to determine if two neighboring superpixels should be merged, is the Chi-squared (*χ* ^2^) histogram distance [7]. The *χ* ^2^ distance between two histograms *P* and *Q* is defined as

3 $$  \chi^{2} (P,Q) = \frac{1}{2}\sum\limits_{k} {\frac{{(P_{k} - Q_{k})^{2} }}{{P_{k} + Q_{k} }}}.  $$

We represent the intensity distribution in each channel of each superpixel as a histogram and use the following formula to measure the similarity distance *D*_*C*_(*i,j*) between superpixels *i* and *j*,

4 $${} \begin{aligned} &D_{C}(i,j) =\\ &\quad \sqrt {\left({\chi^{2} ({H_{i}^{c}},{H_{j}^{c}})} \right)^{2} + \left({\chi^{2} ({H_{i}^{y}},{H_{j}^{y}})} \right)^{2} + \left({\chi^{2} ({H_{i}^{r}},{H_{j}^{r}})} \right)^{2} }, \end{aligned}  $$

where *H* ^*c*^,*H* ^*y*^,*H* ^*r*^ are the superpixel histograms of channels cyan, yellow, and red, respectively.

In addition to *D*_*C*_(*i,j*), another measure called *edge strength* is used for the superpixel merging decision. The edge strength *E*_*ij*_ between superpixels *i* and *j* is defined as the average edge probability over all the pixels in superpixel *i* having at least one neighbor belonging to superpixel *j*. The *D*_*C*_ and edge strength are then used in a series of thresholds. If superpixels *i* and *j* have similar colors, it might be because they are part of the same fiber, or it might be because they come from different fibers that happen to have similar colors. Therefore, whenever two superpixels have smilar *D*_*C*_ (the *χ* ^2^ distance is not greater than a threshold *thres*_*dC*), they can only be merged if they have low edge strengths (their edge strengths are not greater than a threshold *thres*_*edge*). In addition, we use another predefined threshold *thres*_*size* to avoid muscle fibers having unrealistic sizes which are formed from over-merging. However, this size limitation is not applied to superpixels representing “background” (namely, black-colored superpixels with mean color intensity below *tres*_*bg*).

**Fig. 8:**
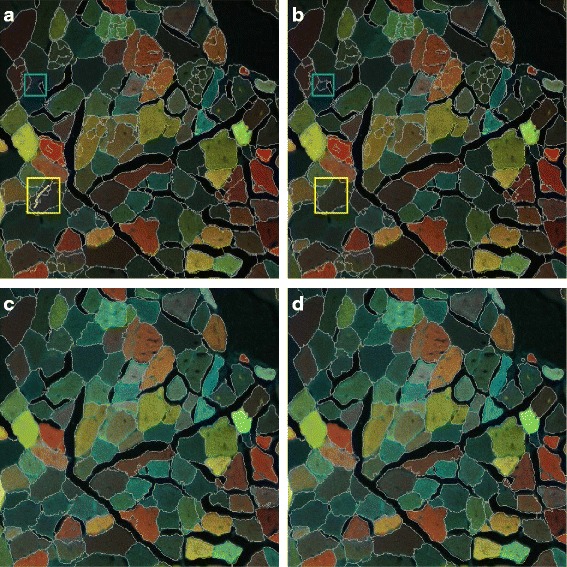
Segmentation using superpixels. (**a**) Automatic superpixel grouping; (**b**) Example of manual refinement: *yellow box* - draw a curve to merge all the regions along the curve, *blue box* - restore a superpixel to merge it with a neighboring region in another way; (**c**) Final result; (**d**) Ground truth

Our iterative superpixel merging algorithm starts with a calculation of the *χ* ^2^ distances and the edge strengths between each superpixel and its neighbors. Then the method for merging superpixels is a series of thresholded criteria as described in Fig. 5. The algorithm stops when there are no more superpixels merged. Figure 8 a shows a result after this processing step.

### Manual refinement

For challenging datasets, it is impossible to produce an error-free segmentation. Errors from the automatic superpixel merging process include two types:

1. Over-merging: merging of superpixels from different muscle fibers, or from a muscle fiber and neighboring background/artifacts.
2. Under-merging: some neighboring superpixels from the same muscle fiber have not been merged yet.

A manual refinement step is introduced to our framework through a user-friendly GUI in order to fix the superpixel merging errors (Fig. 8 b). The main supported operations include

- Drawing a freehand curve to merge all the superpixels or regions along the curve.
- Restoring the original superpixels surrounding a selected position to allow manually merging them in another way.

If the superpixel generation produces a high boundary recall, using these two operations can guarantee a very good segmentation result (Fig. 8 c).

After this manual refinement, the superpixel segmentation is combined with the nuclear segmentation in a postprocessing step to form the final segmentation result.

## Results

### Superpixel evaluation

We use two error metrics, *boundary recall* and *under-segmentation error*, to evaluate our SLIC-MMED algorithm and compare it with the original SLIC and another advanced superpixel algorithm named VCells [8]. Boundary recall is the fraction of ground truth edges that fall within a certain distance *d* (*d*=2 in our experiments) of at least one superpixel boundary. A good superpixel segmentation should produce a high boundary recall. Under-segmentation error compares segment areas to measure to what extend superpixels flood over the ground truth segment borders. Details about the calculation of these two measures can be found in [4].

We used the dataset in Fig. 2 for this evaluation. The corresponding ground truth was created by one computer expert under the supervison of our muscle biology expert (Fig. 8 d). The compactness was chosen as 20 for all the three algorithms. The edge weight *α* in Eq. 2 was 20 for SLIC-MMED. The input image for the original SLIC and VCells algorithms was the composite color image from the cyan, yellow and red channels after preprocessing (Fig. 2 f).

**Fig. 9:**
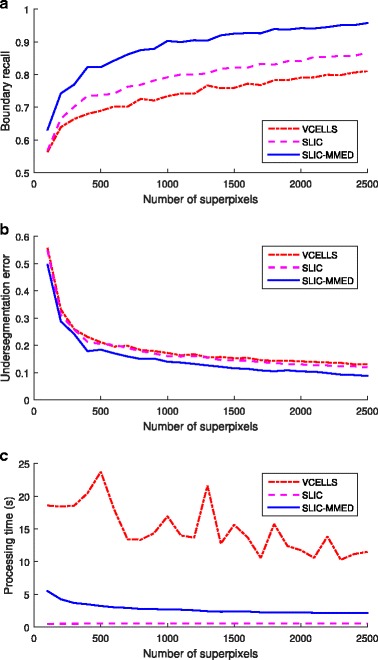
Comparison of different superpixel methods using different measures: (**a**) Boundary recall; (**b**) Under-segmentation error; (**c**) Processing time. All algorithms were run repeatedly with the number of superpixels ranging from 100 to 2500 with a step size of 100. The computing platform was a 3.50GHz Intel Xeon CPU E3-1246 desktop computer with 32 GB RAM running Microsoft Windows 7 Professional 64-bit

Figure 9 shows the performance of the three superpixel algorithms, scored using boundary recall, under-segmentation error and processing time (in seconds). As can be seen, our proposed SLIC-MMED outperforms the other two algorithms in term of superpixel quality. In term of processing time, our algorithm is slower than the original SLIC due to the extras processes, but is still very fast (about 2–5 seconds for a 1024×1024-pixel three-channel image) and much faster than VCells. Algorithmic efficiency is not a limiting factor in our context because the slowest step of our pipeline, preparing tissue sections for microscopy, is much slower than any of the image analysis algorithms.

Figure 7 shows the superpixels generated when each of the candidate algorithms was run on the same image (using a moderate parameter value of 800 for the number of superpixels). The superpixel shapes generated by SLIC-MMED look less regular than those of the other two algorithms due to the introduction of an edge map. However, the superpixels created by SLIC-MMED adhere more closely to the image boundaries, so they have virtually no significant errors. Significant errors are annotated by small white arrows on the images.

### Segmentation evaluation

The final stage of evaluation considers the total sementation accuracy. The methods SLIC and VCells provide only superpixels, not segmentation, so they are not covered in this section. Our method, SLIC-MMED, merges superpixels to create segmented regions, so the quality of its segmentation is inherently related to the quality of its superpixels. We assess the segmentation using an absolute score, defined with respect to ground truth, called the Dice similarity coefficient (DSC) [9]. The DSC measures the spatial overlap between two segmentations, *X* and *Y*, and is defined as

5 $$  DSC = \frac{{2\left| {X \cap Y} \right|}}{{\left| X \right| + \left| Y \right|}},  $$

where |*X*| and |*Y*| are the number of pixels in *X* and *Y*, respectively. It should be noted that Eq. 5 is for the evaluation of one resulting segment. Our segmentation problem is a multiple-object segmentation with multiple fibers and other regions. We propose the median DSC (*medDSC*) which is computed as in Eq. 6 to measure the similarity between the segmentation result *S* and the ground truth *G*.

6 $$  \begin{array}{l} medDSC = \mathop {median}\limits_{i = 1,..,N_{G}} \left({DSC_{i}} \right), \\ DSC_{i}={\frac{{2\left| {g_{i} \cap s_{f(i)}} \right|}}{{\left| {g_{i}} \right| + \left| {s_{f(i)}} \right|}}}, \end{array}  $$

where *N*_*G*_ and *N*_*S*_ are the respective total number of segments in *G* and *S,g*_*i*_ is one segment in *G*, and *s*_*f*(*i*)_ with *f*(*i*)∈[1,*N*_*S*_] is the corresponding segment in *S* having the largest overlap with *g*_*i*_.

For the example data set (Fig. 2), we used SLIC-MMED to generate 1500 superpixels (with compactness = 20 and *α*=20). After the automatic superpixel merging step (Fig. 8 a), we manually refined the segmentation (Fig. 8 b) and got the final result as in Fig. 8 c. The resulting *medDSC* was 0.92 for this dataset.

**Fig. 10:**
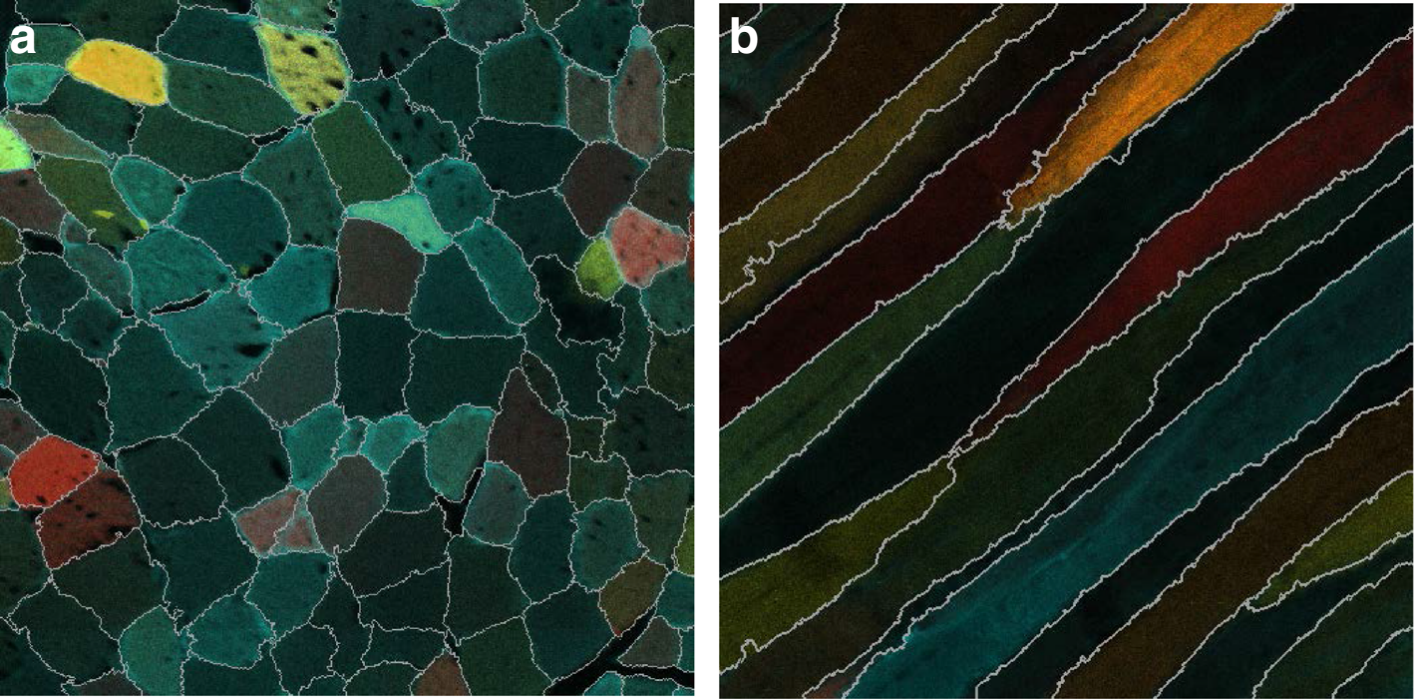
Segmentation results applying SLIC-MMED to the images in Fig. 1. The formats of each image channel of the data are (**a**) 8-bit 1024 × 1024 pixels and (**b**) 12-bit 512 × 512 pixels. Each segmentation is displayed on the composite color image from the *cyan*, *yellow* and *red* channels after preprocessing

Figure 10 presents the final SLIC-MMED segmentation results for the images from Fig. 1. After the superpixel merging phase and prior to the refinement phase, the scores were *medDSC* 0.72 and 0.67 for the cross-section dataset and the lateral-section dataset, respectively. The lower score for lateral sections reflects the extreme aspect ratio (non-compactness) of the underlying objects. Fortunately, the under-segmentation of a fiber into fiber segments is nearly instantaneous for manual refinement to merge correctly. After the refinement phase, the *medDSCs* were 0.93 and 0.95 for the cross-section dataset and the lateral-section dataset, respectively. As recommended by Zijdenbos et al. [10], a segmentation is considered good if *DSC*>0.70.

We also propose *DSC*_*X*_, where *X* is a number between 0 and 100, to measure the fraction of segments in the ground truth are segmented with a *DSC*≥*X*/100. The final segmentation in Fig. 8 c has *DSC*_75_ of 0.78, meaning that 78% of the segments were segmented with a *DSC*≥0.75. For the cross-section dataset and the lateral-section dataset in Fig. 10, the scores were *DSC*_75_ 0.93 and 0.91, respectively.

## Discussion and conclusion

In our experiments, the proposed method can correctly segment muscle fibers in very heterogeneous sections having both bright and dark regions, a wide range of fiber sizes, homogeneous red or yellow but more irregular cyan segments in cytoplasm. The fact that the method can handle a variety of cell sizes and morphologies in these confetti-fluorescent images suggests that it may be useful for analyzing confetti-fluorescent images in other tissues.

With an accurate segmentation, we can count the number of muscle fibers that contain each of the confetti colors. The method works well for even weakly fluorescent areas, as in Figs. 1 a and 10 a. Using the same segmentation, we also can measure the diameter and cross sectional area for each fiber. In most labs, measuring the diameter and cross-sectional area would require cutting a set of adjacent tissue sections, which would then undergo labor-intensive staining, followed by imaging and registration of the adjacent sections, to provide a superpositioning of the stained section and the confetti-labelled section. Staining requires doubling the number of sections because staining eliminates the endogenous fluorescence. In this work we show it is possible to obtain segmentation from the endogenous fluorescence, allowing us to skip the costly process of staining.

In fluorescence microscopy, each fluorophore emits light over a range of wavelengths (its emission spectrum), causing nearby colors to overlap. For example, the emission spectrum of green overlaps with the emission spectrum of yellow. It is for this reason that multi-color labelling strategies have engineered the similar fluorescent proteins to have different sub-cellular localizations (e.g., nuclear localization of the green and cytosolic localization of the yellow). Localization allows the identity of the label to be disambiguated. In our images, the green signal bled into the yellow channel (see Fig. 2 c). SLIC-MMED includes explicit management of different subcellular localizations, and this may be why we had no segmentation errors due to green-yellow spectral overlap.

The observed colors arise from fluorescent protein molecules that are diffusible in their compartment (cytosol, membrane, or nucleus). The Brownian nature of diffusion suggests that color distribution might be nearly uniform across the space of the relevant compartment. In other words, the limit of molecular diffusion becomes the boundary of the color, which we identify as the boundary of the segmentation. Cell membranes are 2-dimensional surfaces which can appear as 1-dimensional curves when imaged from a cross-section. The cytosol and nucleus of a cell are 3-dimensional compartments, which appear as 2-dimensional continuous regions. Edge detection is a natural approach for analyzing 2-dimensional membrane-targeted fluorescence, such as the cyan channel in our images. Meanwhile, superpixel-based region detection methods are a natural approach for analyzing 3-dimensional compartments. If the spatial distribution of the fluorescent proteins were punctate (0-dimensional) or fibrillar (1-dimensional), then our method would be less appropriate.

Our segmentation framework, SLIC-MMED, is a “hybrid” method that combines the advantages of a region-based clustering algorithm (SLIC) and an edge detector through the integrated edge map. The introduction of an user-friendly superpixel refinement module provide flexibility for the framework. As long as the superpixel generation provides a high performance in boundary recall, the framework provides very good segmentations. Our experimental results show a high degree of agreement with experts. In the final scoring, the differences between different trials are also heavily dependent on the specific dataset, the number of superpixels to be revised, and the user’s expertise at performing the manual refinement step. In future, we will intensively analyze the contribution of automation to the effectiveness of the framework.

The algorithm is potentially applicable to other multi-channel microscopy applications besides muscle. Mouse transgenes with confetti, brainbow or other multi-color stochastic labels have become extremely popular [1] and the scientific community is rapidly generating multi-channel images that require analysis. Our image analysis method is particularly valuable for such applications.

## Additional file

Additional file 1 The MATLAB and C code, and the data files used in this project. (ZIP 9346 kb)
